# Serological immunity against vaccine‐preventable diseases in children with inflammatory bowel disease at diagnosis

**DOI:** 10.1002/jpr3.70146

**Published:** 2026-01-27

**Authors:** Clara Noble, Renato Gualtieri, Klara Posfay‐Barbe, Nathalie Rock, Geraldine Blanchard‐Rohner

**Affiliations:** ^1^ Faculty of Medicine University of Geneva Geneva Switzerland; ^2^ Research Platform of Pediatrics, Gynecology, and Obstetrics, Department of Pediatrics, Gynecology and Obstetrics Geneva University Geneva Switzerland; ^3^ Unit of Infectious Diseases, Division of General Pediatrics, Department of Pediatrics, Gynecology and Obstetrics Geneva University Hospitals and University of Geneva Geneva Switzerland; ^4^ Unit of Gastroenterology, Hepatology and Nutrition, Division of Pediatric Specialties, Department of Pediatrics, Gynecology and Obstetrics Geneva University Hospitals and University of Geneva Geneva Switzerland; ^5^ Unit of Immunology, Vaccinology, and Rheumatology, Division of General Pediatrics, Department of Pediatrics, Gynecology and Obstetrics Geneva University Hospitals and University of Geneva Geneva Switzerland

**Keywords:** antibody, colitis, infections, pediatric, vaccine

## Abstract

**Objectives:**

Patients with inflammatory bowel disease (IBD) are at increased risk of vaccine‐preventable diseases. However, vaccination coverage in this population is often suboptimal. This retrospective study assessed the vaccination status and vaccine serology of children diagnosed with IBD in a high‐income country with broad vaccine access.

**Methods:**

Medical records of children under 16 years diagnosed with IBD between 2009 and 2023 were retrospectively reviewed. Demographic and clinical data, vaccine history, and vaccine serology, at the time of diagnosis, were analyzed.

**Results:**

A total of 42 IBD patients were included, with a median age at diagnosis of 11 years. Vaccine records were available for 31 (74%), of whom only 6 (19%) had up‐to‐date vaccinations. Vaccine serology did not always correlate with vaccine history. Most patients had the required doses for age for tetanus, diphtheria, *Haemophilus influenzae* Type B (Hib), and measles. In contrast, coverage was lower for Hepatitis B (61%, *n* = 19/31), Hepatitis A (45%, *n* = 14/31), *Streptococcus pneumoniae* (55%, *n* = 17/31), and varicella (32%, *n* = 10/31). Corresponding seroprotection rates varied: 90% for tetanus (*n* = 19/21), 92% for diphtheria (*n* = 12/13), 80% for measles (*n* = 16/20), 76% for varicella (*n* = 19/25), 77% for Hib (*n* = 10/13), but only 22% for *S. pneumoniae* (*n* = 4/18), 54% for Hepatitis B (*n* = 13/24), and 22% for Hepatitis A (*n* = 2/9).

**Conclusions:**

This study reveals major gaps in vaccination coverage and seroprotection among children with IBD. Pneumococcal and Hepatitis A/B vaccines should be given at diagnosis, and measles and varicella serology should be assessed to enable timely catch‐up with live vaccines before immunosuppression.

## Abstract (highlights)

What is Known

- Children with inflammatory bowel disease (IBD) are at increased risk of infections and often require immunosuppressive therapy.
- Vaccination coverage in IBD patients is generally suboptimal.
- Live vaccines (e.g., measles, mumps, and rubella [MMR], varicella) are generally contraindicated after immunosuppression begins.

What is New

• Only 19% of children with IBD had up‐to‐date vaccinations at diagnosis.
• Protection was especially low for Hepatitis A/B and pneumococcus.
• If serologic data and vaccination documentation are lacking, empiric Hepatitis A/B and pneumococcal vaccination should be considered as a practical measure.

## 1 INTRODUCTION

Inflammatory bowel disease (IBD) patients experience immune dysregulation, leading to chronic inflammation. This persistent inflammation causes pain, malabsorption, and various gastrointestinal symptoms while also increasing the risk of malignancies.^1^ To manage inflammation effectively, patients often require the timely initiation of immunosuppressive therapy, including anti‐TNF agents. Regardless of the specific medication used, immunosuppressive treatments in IBD patients have been shown to elevate the risk of opportunistic infections by 3.9‐fold (95% confidence interval [CI], 2.2–6.9), such as pneumococcal and varicella infections. This risk increases further with the use of multiple immunosuppressive therapies.^1^

Ensuring adequate protection against infections in individuals with IBD remains a significant challenge. The 2012 European Society for Paediatric Gastroenterology, Hepatology and Nutrition (ESPGHAN) Porto group commentary, the 2014 ESPGHAN guidance, and the 2021 European Crohn's and Colitis Organisation (ECCO)/ESPGHAN statements all emphasize that vaccination status should be reviewed at the time of diagnosis.^1^, ^2^, ^3^ Additionally, all age‐appropriate vaccines should be administered before initiating immunosuppressive treatments,^2^, ^3^, ^4^, ^5^, ^6^ and, in accordance with Swiss guidelines, varicella and measles serologies “independent of infectious history and vaccination status” should be performed.^3^ However, despite the availability of international guidelines, adherence in routine practice remains low, and vaccination rates among IBD patients are suboptimal.^4^, ^5^, ^6^, ^7^, ^8^ For instance, a study by Melmed et al. in the United States found that only 9% of IBD patients received the pneumococcal vaccine, despite recommendations from the Advisory Committee on Immunization Practices (ACIP).^7^ At our institution, we routinely administer vaccine boosters based on serology results to customize vaccination catch‐up in newly immunocompromised patients. Unfortunately, this approach is not available at most centers. The objectives of our study were therefore to assess the vaccination status and seroprotective levels of children with IBD at the time of diagnosis in a high‐income country with accessible vaccines, national vaccination guidelines, and available vaccine serology testing.

## 2 METHODS

### 2.1 Ethics statement

The study was approved by the Geneva Regional Ethics Committee (CCER 2020‐01537).

### 2.2 Study setting and population

This retrospective observational study was conducted in the Geneva University Hospitals (HUG) (Geneva, Switzerland). Children diagnosed with IBD (Crohn's disease, ulcerative colitis, or others) between 2009 and 2023, and less than 16 years of age at diagnosis, were included in the study. Patients needed to have at least one vaccine serology available to be included.

### 2.3 Data collection

A systematic review of electronic medical records (EMRs) was performed with the Research Electronic Data Capture (REDCap) system to collect demographics, vaccine history, and serological data.

### 2.4 Definition of vaccine status and immunity

Vaccine status was classified as “up‐to‐date” or “not‐up‐to‐date” depending on the Swiss vaccination guidelines for 2023 for each vaccine according to the patient's age (see Table S1).^3^, ^5^ This approach was selected as it most closely resembles clinical practice, where vaccinations are updated at diagnosis to the current schedule.

Patients not yet eligible for a given vaccine by age were therefore considered up‐to‐date. Applying the 2023 vaccination schedule retrospectively, children who received vaccinations between 2009 and 2023 would still meet the criteria for being “up‐to‐date,” limiting misclassification due to changes in the schedule.

Vaccination and history of natural infections were obtained from the patient′s vaccination booklet. This document is filled out by professional healthcare workers and recorded at the hospital.

All vaccine serologies were performed as standard vaccine follow‐up for these patients and analyzed in the vaccinology laboratory at HUG for tetanus, diphtheria, *Haemophilus influenzae* Type B (Hib), pneumococcus, measles, and varicella, and in the virology laboratory for Hepatitis A and B.

Vaccine immunity was defined according to laboratory cut‐offs used at the HUG and is as follows: varicella (150 IU/L), measles (150 IU/L), diphtheria (100 IU/L), tetanus (100 IU/L), Hib (0.15 mg/L), and Hepatitis A and B (10 IU/L).^6^

Regarding pneumococcal immunity, patients with serotype‐specific immunoglobulin G (IgG) levels >0.50 mg/L in response to at least four of the seven tested serotypes (4, 6b, 9 v,14, 18c, 19 F, and 23 F) were considered “seroprotected.”^6^ These were measured with an enzyme‐linked immunosorbent assay^7^ or a multiplex binding assay after 2019.^8^, ^9^

### 2.5 Statistical analysis

Categorical data are presented as frequencies and percentages. Continuous data are reported as medians and interquartile ranges (IQRs), or as geometric mean concentrations (GMC) with 95% CI, where applicable. Due to the descriptive nature of our study, no sample size calculation was performed; instead, all patients at our center who met the inclusion criteria were included. Statistical analyses and associated graphs were conducted using Stata version 17 (2021; StataCorp), GraphPad Prism version 6.04 for Windows (GraphPad Software, www.graphpad.com), or RStudio (Posit Team, 2025, www.posit.co).

## 3 RESULTS

### 3.1 Patient characteristics

In this study, 42 patients were included; their characteristics are reported in Table 1. The median age of the patients at diagnosis was 11 (IQR 5) years. Among the 42 included patients, 41% were female. The majority were from Europe (*n* = 32, 76%). Crohn's disease was the most frequent type of IBD (*n* = 25, 60%), followed by ulcerative colitis (*n* = 10, 24%). For additional information on subsequent treatment introduced after the diagnosis, see Table S2.

**Table 1:** Demographic, clinical characteristics, and vaccine status of 42 included patients.

Age at diagnosis in years, median [IQR]	11 [5]
Female, *n* (%)	17/42 (41)
Origin by continent, *n* (%)	
Africa	3/42 (7)
North America	1/42 (2)
South America	3/42 (7)
Asia	3/42 (7)
Europe	32/42 (76)
IBD type, *n* (%)	
Crohn's disease	25/42 (60)
Ulcerative colitis	10/42 (24)
Undetermined IBD	7/42 (17)
Vaccine status at diagnosis, *n* (%)	
Fully up‐to‐date for age	6/31 (19)
Not fully up‐to‐date for age	25/31 (81)
Unknown	11/42 (26)
Distribution of lack of immunization, *n* (%)^a^	
Not up‐to‐date for one vaccine target	4/31 (13)
Not up‐to‐date for two vaccine targets	3/31 (10)
Not up‐to‐date for three vaccine targets	5/31 (16)
Not up‐to‐date for more than three vaccine targets	13/31 (42)
IgG level at diagnosis (95% CI)^b^	11.47 (9.87, 13.07)

Abbreviations: CI, confidence interval; IBD, inflammatory bowel disease; IgG, immunoglobulin G; IQR, interquartile range.

^a^ For patients with available vaccine records.

^b^ Available for 28 patients.

### 3.2 Vaccine history

Among the 42 patients, 31 (74%) had available vaccine records (see Figure S1). When vaccine records were available, 6/31 (19%) patients had fully up‐to‐date vaccination for age, and 25/31 (82%) had not fully up‐to‐date vaccinations (Tables S1 and S3).

### 3.3 Vaccine immunity

**Figure 1:**
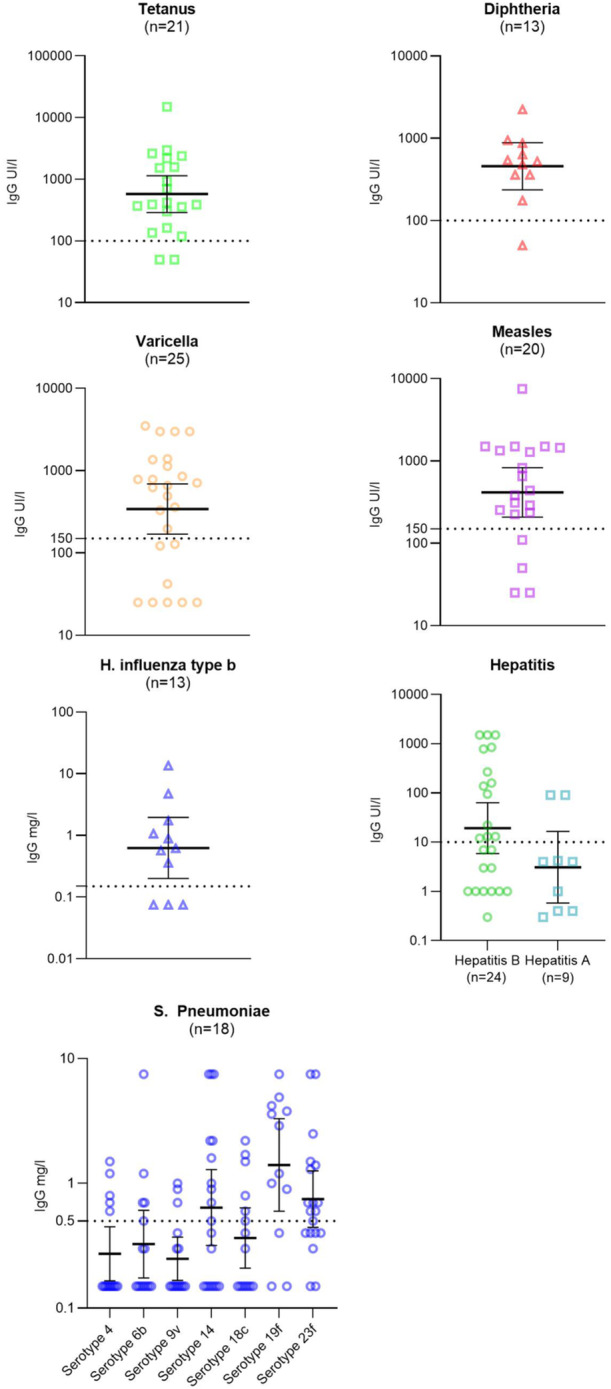
Antigen specific IgG levels of the 42 included patients against vaccine‐preventable diseases at diagnosis before any booster. Each symbol represents an individual patient's serological value. The dotted line marks the immunity threshold according to the disease type, and error bars indicate the 95% confidence intervals. Serological results were available for the following antigens: varicella (25/42, 60%), measles (20/42, 48%), diphtheria (13/42, 31%), tetanus (21/42, 50%), *Haemophilus influenzae* Type B (13/42, 31%), Hepatitis B (24/42, 57%), Hepatitis A (9/42, 21%), and *Streptococcus pneumoniae* (18/42, 43%). IgG, immunoglobulin G.

At the time of IBD diagnosis, among patients with available vaccine serology, seroprotection rates were as follows: 19 out of 25 (76%) for varicella, 16 out of 20 (80%) for measles, 12 out of 13 (92%) for diphtheria, and 19 out of 21 (90%) for tetanus. Additionally, among children younger than 5 years, 10 out of 13 (77%) were seroprotected against Hib. Seroprotection rates for Hepatitis B, Hepatitis A, and *Streptococcus pneumoniae* were 13 out of 24 (54%), 2 out of 9 (22%), and 4 out of 18 (22%), respectively (see Table 2, Figure 1).

**Table 2:** Number of seroprotected patients at diagnosis before any booster according to vaccine target among the 42 included patients.

Vaccine antigen	Up to date for age,^a^ *n* (%)^b^	Patients with available serologies, *n* (%)^c^	Total seroprotected, *n* (%)^d^	Seroprotected among up‐to‐date for age^a^), *n* (%)	Seroprotected among not up‐to‐date for age,^a^ *n* (%)	Seroprotected among patients without available vaccine history
Varicella	10/31 (32)	25/42 (60)	19/25 (76)	5/6 (83)	7/11 (65)	7/8 (88)
Measles	26/31 (84)	20/42 (48)	16/20 (80)	11/14 (79)	2/2 (100)	3/4 (75)
Diphteria	23/31 (74)	13/42 (31)	12/13 (92)	8/8 (100)	2/2 (100)	2/3 (67)
Tetanus	23/31 (74)	21/42 (50)	19/21 (90)	11/13 (85)	2/2 (100)	6/6 (100)
*Haemophilus influenzae* Type b	24/31 (77)	13/42 (31)	10/13 (77)	6/9 (67)	1/1 (100)	3/3 (100)
Hepatitis B	19/31 (61)	24/42 (57)	13/24 (54)	4/9 (44)	2/5 (40)	7/10 (70)
Hepatitis A	14/31 (45)	9/42 (21)	2/9 (22)	1/4 (25)	0/1 (0)	1/4 (25)
*Streptococcus pneumoniae*	17/31 (55)	18/42 (43)	4/18 (22)	2/10 (20)	1/3 (33)	1/5 (20)

*Note*: This table summarizes, for each vaccine, the proportion of patients who were up to date for age at the time of diagnosis, according to the 2023 Swiss National Immunization Schedule, the proportion with available serology results, and the proportion demonstrating seroprotection at the time of diagnosis. Among the 42 patients included in the study, serologic data were available for those indicated in Column 2, while vaccination records were accessible for 31 patients. Serologic protection at diagnosis was then analyzed according to vaccination status: up to date, not up to date, and patients without documented vaccination history.

^a^ “Up to date for age” is defined according to Swiss recommendations among patients with available vaccine information for each vaccine (see Table 1).

^b^ Among the 31 patients with available vaccination records.

^c^ Among the 42 included patients with available serology results.

^d^ Seroprotection is defined according to antigen‐specific threshold levels.

Among children up to date for age, seroprotection was observed in 5 out of 6 (83%) patients for varicella, 11 out of 14 (79%) for measles, 8 out of 8 (100%) for diphtheria, 11 out of 13 (85%) for tetanus, 6 out of 9 (67%) for Hib, 4 out of 9 (44%) for Hepatitis B, 1 out of 4 (25%) for Hepatitis A, and 2 out of 10 (20%) for *Streptococcus pneumoniae*.

Among those not up to date for age, seroprotection was 7 out of 11 (65%) for varicella, 2 out of 2 (100%) for measles, 2 out of 2 (100%) for diphtheria, 2 out of 2 (100%) for tetanus, 1 out of 1 (100%) for *H. influenzae* Type b, 2 out of 5 (40%) for Hepatitis B, 0 out of 1 (0%) for Hepatitis A, and 1 out of 3 (33%) for *S. pneumoniae*.

Among patients without available vaccination history, seroprotection was 7 out of 8 (88%) for varicella, 3 out of 4 (75%) for measles, 2 out of 3 (67%) for diphtheria, 6 out of 6 (100%) for tetanus, 3 out of 3 (100%) for Hib, 7 out of 10 (70%) for Hepatitis B, 1 out of 4 (25%) for Hepatitis A, and 1 out of 5 (20%) for *S. pneumoniae* (see Table 2, Figure 1).

## 4 DISCUSSION

This retrospective study of 42 IBD patients with available vaccine serology suggests that baseline vaccine coverage and seroprotection were suboptimal in most cases. This underlines the necessity to carefully review vaccination history and serology prior to administration of immunosuppressive therapies, in accordance with established guidelines.^1^, ^2^, ^3^

Discordance between documented vaccination history and serological protection has been reported previously; our findings confirm this observation in a Swiss pediatric IBD cohort. Serological immunity to varicella, diphtheria, and tetanus was higher than expected based on vaccination records, whereas immunity to Hepatitis B, Hepatitis A, and *S. pneumoniae* was lower despite up‐to‐date vaccination history. Ford et al. have reported similar discrepancies between vaccination history and serological immunity at diagnosis in pediatric patients with a new diagnosis of IBD.^10^ It is possible that chronic inflammation or malnutrition may affect vaccine responses or increase the loss of vaccine antibodies.^11^, ^12^ However, no studies have evaluated the rate of vaccine antibody loss in patients with autoimmune diseases such as IBD compared to healthy controls.

Consistent with published guidance, we support assessing Hepatitis A/B and pneumococcal immunity at diagnosis and providing catch‐up vaccination according to national guidelines. When serologies or vaccination records are unavailable, empiric vaccination against Hepatitis A/B and pneumococcus may be considered as a pragmatic approach. While many IBD centers have access to serological testing, these issues are particularly relevant for centers facing barriers such as cost or logistical constraints, underscoring the need for practical solutions. Regarding Hepatitis B, current guidelines recommend maintaining an antibody level of at least 10 IU/L in immunosuppressed patients to ensure adequate protection.^3^ It is well established that anti‐HBs antibody levels naturally decline over time, even when vaccination has induced protective memory immunity. In immunosuppressed patients, however, this memory response may be insufficient to rapidly mount an antibody response and neutralize the virus upon exposure.^13^ Our findings highlight the importance of assessing Hepatitis B immunity at diagnosis to identify patients who may require catch‐up vaccination. This should be interpreted alongside recent data from Ulrich et al.,^13^ which indicate that although antibody titers wane over time, breakthrough infections remain rare, suggesting that routine periodic serological testing after complete vaccination may not be necessary.

We found that almost one‐third of the patients were not protected against varicella or measles. Since these live vaccines are generally contraindicated once immunosuppression has begun, it is crucial to monitor varicella and measles serologies as soon as an autoimmune disease like IBD is suspected. This allows for timely catch‐up vaccination with measles, mumps, and rubella (MMR) and varicella zoster virus (VZV) vaccines at diagnosis, provided there is an opportunity to delay immunosuppressive treatment for at least 4 weeks. It is to note that new research by Keutler et al. suggests that in selected stable patients, vaccination with MMR vaccination could still be considered.^14^ Indeed, they reported no serious adverse events and demonstrated measurable immune responses in their 22 selected, stable patients receiving low or moderate immunosuppression. While these findings are reassuring, live‐attenuated vaccination should still be undertaken with caution, ideally before initiating or escalating immunosuppressive therapy, and only in patients with low‐level or stable immunosuppression after appropriate immunologic assessment.^15^

Our study identifies key areas for improving protection in this vulnerable population. Notably, vaccine records (copies of the vaccination booklets) were missing from the EMRs for a quarter of patients, likely because they were not uploaded to our EMRs. The retrospective study design precluded direct patient contact, limiting our ability to obtain complete vaccination records. Access to accurate vaccination history is crucial for developing appropriate immunization plans tailored to each patient. To address this gap, optimizing the use of EMRs has been proposed as a strategy to enhance record standardization and ensure continuity of care.^16^, ^17^ National registries contribute to improving quality‐of‐care indicators and could serve as a cornerstone for enhancing guideline adherence, particularly in documenting vaccinations and assessing the need for boosters.^18^ In Switzerland, there are currently no such registries. Additionally, an alert system could be implemented to prompt healthcare providers to collect this data when considering the prescription of immunosuppressive drugs. While this recommendation is based on findings from the pediatric IBD context, it supports broader public health efforts to enhance vaccination rates among at‐risk populations.

Secondly, when vaccine records were available, there was often a lack of vaccination, especially for pneumococcus, varicella, and Hepatitis A and B. This observation is in line with previous studies,^19^, ^20^ including in the Swiss adult IBD patients,^21^ where most patients did not receive adequate doses of vaccines for age for almost all scheduled vaccines. In the case of Hepatitis A, low vaccination rates can be explained by the fact that, in the routine vaccination schedule, it is recommended only for individuals traveling to endemic regions or those with liver disease. Additionally, the observed coverage patterns must be interpreted in the context of evolving recommendations. Lower coverage for vaccines such as Meningococcus or HPV may reflect the timing of guideline changes, rather than poor adherence. In patients with incomplete or uncertain vaccination status, empiric booster doses may be considered to ensure adequate immunity. When feasible, vaccination should ideally be completed before initiating or escalating immunosuppressive therapy to maximize safety and immune response. In cases where prior records are unavailable or immunity is uncertain, full re‐immunization according to national guidelines may be warranted. The risk‐benefit balance of delaying treatment must be carefully considered on an individual basis, prioritizing disease control while optimizing protection against vaccine‐preventable infections.

Our study has several limitations: its retrospective design limits the ability to collect all relevant data, particularly regarding vaccination history and serology results. Additionally, the relatively small sample size reduces the statistical significance of the findings and allows to provide only descriptive data. Furthermore, since this study was conducted in a single tertiary center, without a control group of healthy children, this limits our ability to fully assess the influence of chronic inflammation and nutritional status on vaccine responses.

## 5 CONCLUSION

Despite the limitations of this study, the low documented vaccination coverage and suboptimal immunity in this cohort highlight the need for quality improvement measures to ensure pediatric IBD patients receive guideline‐recommended vaccinations. Further prospective studies are needed to confirm these findings and link coverage to clinical outcomes. Such studies should investigate factors affecting long‐term seroprotection, including demographic characteristics, IBD subtype, immunosuppressive therapy, vaccination timing, and the interplay between coverage, immunity, and disease onset. Incorporating a control group would clarify the impact of inflammation and immunosuppression on vaccine responses. Additionally, identifying barriers to vaccination uptake is essential for developing targeted and effective interventions.

## CONFLICT OF INTEREST STATEMENT

The authors declare no conflicts of interest.

## Supporting information

Supplementary figure S1.

Supplementary Table S1_140126.

Supplementary Table S2.

Supplementary Table S3_140126.
