# Supplementary material for: Serological immunity against vaccine‐preventable diseases in children with inflammatory bowel disease at diagnosis
Source: JPGN Rep. 2026 Jan 27;7(2):289–95. doi: 10.1002/jpr3.70146 (PMC13150987; doi:10.1002/jpr3.70146)
Supplement: Supplementary file 1 — Supplementary figure S1. [file JPR3-7-289-s004.docx]

Supplementary Figure 1: Study population flowchart
