# Supplementary material for: Serological immunity against vaccine‐preventable diseases in children with inflammatory bowel disease at diagnosis
Source: JPGN Rep. 2026 Jan 27;7(2):289–95. doi: 10.1002/jpr3.70146 (PMC13150987; doi:10.1002/jpr3.70146)
Supplement: Supplementary file 3 — Supplementary Table S2. [file JPR3-7-289-s002.docx]

Supplementary Table 2 : IBD treatment plan instituted after diagnosis

*Legend : This table provides details on the treatments received by our cohorts, offering additional information about our patients.*

| IBD treatment plan, n (%) |  |
| --- | --- |
| Combination: Anti-TNF-α (Infliximab) ± Azathioprine ± 5-Aminosalicylic Acid (5-ASA) ± Prednisone | 29/42 (69) |
| Azathioprine | 4/42 (10) |
| Anti-TNF-α (Adalimumab) ± Azathioprine | 3/42 (7) |
| 5-Aminosalicylic Acid (5-ASA) + Azathioprine | 1/42 (2) |
| Prednisone + Azathioprine | 1/42 (2) |
| Vedolizumab ± Azathioprine | 1/42 (2) |
| Ustekinumab ± Azathioprine | 1/42 (2) |
| Abatacept | 1/42 (2) |
