# Supplementary material for: Serological immunity against vaccine‐preventable diseases in children with inflammatory bowel disease at diagnosis
Source: JPGN Rep. 2026 Jan 27;7(2):289–95. doi: 10.1002/jpr3.70146 (PMC13150987; doi:10.1002/jpr3.70146)
Supplement: Supplementary file 4 — Supplementary Table S3_140126. [file JPR3-7-289-s003.docx]

Supplementary Table 3: Vaccine status at diagnosis for each vaccine preventable disease for the 31 patients with available vaccine records

| **n (%)** | **Varicella** | **Measles, Mumps, Rubella** | **Diphtheria** | **Tetanus** | **Pertussis** | **Haemophilus influenzae type B** | **Polio** | **Hepatitis B** | **Hepatitis A** | **S. pneumoniae** | **Human Papillomavirus** | **Meningococcus** |
| --- | --- | --- | --- | --- | --- | --- | --- | --- | --- | --- | --- | --- |
| Up-to-date for Age* | 10 (32) | 26 (84) | 23 (74) | 23 (74) | 23 (74) | 24 (77) | 25 (81) | 19 (61) | 14 (45) | 17 (55) | 21 (68) | 21 (68) |
| Not up-to-date for Age* | 21 (68) | 5 (16) | 8 (26) | 8 (26) | 8 (26) | 7 (23) | 6 (19) | 12 (39) | 17 (55) | 14 (45) | 10 (32) | 10 (32) |

* According to Swiss recommendations amongst patients with available vaccine information for each vaccine (see Table 1)
